# Immune enhancement by novel vaccine adjuvants in autoimmune-prone NZB/W F1 mice: relative efficacy and safety

**DOI:** 10.1186/1471-2172-12-61

**Published:** 2011-10-24

**Authors:** Youssef Aachoui, Swapan K Ghosh

**Affiliations:** 1 1Department of Biology, Indiana State University, Terre Haute, IN 47809, USA

## Abstract

**Background:**

Vaccines have profoundly impacted global health although concerns persist about their potential role in autoimmune or other adverse reactions. To address these concerns, vaccine components like immunogens and adjuvants require critical evaluation not only in healthy subjects but also in those genetically averse to vaccine constituents. Evaluation in autoimmune-prone animal models of adjuvants is therefore important in vaccine development. The objective here was to assess the effectiveness of experimental adjuvants: two phytol-derived immunostimulants PHIS-01 (phytanol) and PHIS-03 (phytanyl mannose), and a new commercial adjuvant from porcine small intestinal submucosa (SIS-H), relative to a standard adjuvant alum. Phytol derivatives are hydrophobic, oil-in water diterpenoids, while alum is hydrophilic, and SIS is essentially a biodegradable and collagenous protein cocktail derived from extracellular matrices.

**Results:**

We studied phthalate -specific and cross-reactive anti-DNA antibody responses, and parameters associated with the onset of autoimmune disorders. We determined antibody isotype and cytokine/chemokine milieu induced by the above experimental adjuvants relative to alum. Our results indicated that the phytol-derived adjuvant PHIS-01 exceeded alum in enhancing anti-phthalate antibody without much cross reactivity with ds-DNA. Relatively, SIS and PHIS-03 proved less robust, but they were also less inflammatory. Interestingly, these adjuvants facilitated isotype switching of anti-hapten, but not of anti-DNA response. The current study reaffirms our earlier reports on adjuvanticity of phytol compounds and SIS-H in non autoimmune-prone BALB/c and C57BL/6 mice. These adjuvants are as effective as alum also in autoimmune-prone NZB/WF1 mice, and they have little deleterious effects.

**Conclusion:**

Although all adjuvants tested impacted cytokine/chemokine milieu in favor of Th1/Th2 balance, the phytol compounds fared better in reducing the onset of autoimmune syndromes. However, SIS is least inflammatory among the adjuvants evaluated.

## Background

Prophylactic vaccination is considered the most cost-effective way to control diseases; however, in recent years, there has been growing doubts about the benefits of vaccines, primarily because of largely unsupported claims that constituents in vaccine formulations may have long-lasting deleterious effects. These concerns have led to a surge of efforts to redesign vaccines by employment of modern technologies involving recombinant protein antigens, purified allergens, and pathogen-associated offending agents [1]. Alongside, there are new efforts directed to molecularly defined adjuvants or immunostimulants that nonspecifically boost immunogenic potentials of a vaccine. Once considered "immunologists' dirty tricks", adjuvants are garnering considerable attention with regard to their modes of action, safety, and effectiveness. A major focus is to overcome the constraints of empiricism in the choice of adjuvants and develop efficacious vaccines for populations with varying degrees of immune competence.

To develop safe and broadly effective immunostimulants from structurally diverse compounds, ranging from bacterial products and inorganic salts to biosynthetic intermediates and proteins, is a technical challenge [2]. In an effort to address the issue, we focused on the phytol component of chlorophyll and studied different phytol derivatives for adjuvanticity [3,4]. Although phytol, a diterpenoid related to vitamin E, is known for many beneficial effects in animal studies, it could also be toxic as an adjuvant at high doses [5,6]. In earlier studies, we observed that modified phytol compounds such as PHIS-01 (Phytanol) and PHIS-03 (Phytanyl mannose) are safe and highly effective adjuvants in immunocompetent inbred strains of mice, BALB/c and C57BL/6 [3,4,7,8]. They enhance immunogenicity of many soluble protein antigens and also of heat-killed pathogens [3,4,7,8]. In some instances, phytol compounds work better than alum, the widely used adjuvant licensed for human usage. Arguably, not all vaccine recipients are equally immunocompetent. This necessitates an evaluation of putative adjuvants alone and in combination with vaccine materials in both normal and compromised subjects.

This study focused on autoimmune-susceptible NZB/W F1mice strains that develop renal pathology, circulating immune complexes and auto-antibodies like anti-ds-DNA antibodies. In these mice, immune complexes get deposited in the glomerulus and incite strong immunological and inflammatory responses characterized by production of pro-inflammatory cytokines and chemokines, recruitment and activation of circulating leukocytes, and tissue damage. Despite being immune enhancers, adjuvants could also cause aggravation of autoimmune disorders. An isoprenoid adjuvant, pristane, has been shown to promote lupus-like syndromes and pathologic nephritis in both autoimmune-prone and non-susceptible mouse strains after a single intra-peritoneal administration [9-11]. This is in contrast to the effects of isoprenoids phytol and its derivative PHIS-01[3]. Furthermore, squalene, a triterpene and Freunds' adjuvants (CFA/IFA) could also provoke lupus-like syndromes in non autoimmune- prone BALB/c mice [12]. Obviously these adjuvants in a vaccine would likely be harmful in genetically predisposed or environmentally compromised individuals. In this context, not only phytol, but also its derivatives like PHIS-01 have been found safer [3]. Whether this is true for PHIS-03 (phytanyl mannose), which by virtue of its composition is less hydrophobic than PHIS-01, is not known.

Another experimental adjuvant, SIS (porcine small intestinal submucosa) is a collagenous extracellular matrix (ECM) preparation from Cook biotech, that is licensed for use in human and utilized widely as a non-toxic scaffolding biomaterial in wound healing [13-17]. In many studies, including ours, SIS proved to be a highly effective adjuvant in immunocompetent mice strains [18]. Since SIS contains evolutionarily conserved proteins such as collagen and traces of other proteins of ECM, it can be regarded as a cocktail of adjuvants. However, how effective it is in autoimmune prone mice NZB/WF1 has not been previously addressed.

In an earlier report, we established that phthalate, which is a plasticizer often used in medical devices and as a solvent in cosmetics, can induce cross reactive anti-dsDNA antibody response in both non autoimmune prone mice (BALB/c) as well susceptible NZB/WF1 mice [19-21]. However, only NZB/WF1 mice develop aggravated lupus-like syndromes characterized by high levels of antibodies, renal pathology and considerable mortality rates [19-21]. In this report, we used phthalate-KLH conjugate as the immunogen in NZB/WF1 mice with or without alum or the experimental adjuvants SIS, PHIS-01 and PHIS-03. This study was undertaken to examine immune-modulatory changes inflicted by choice of adjuvant, which may either suppress or aggravate the autoimmune syndromes in NZB/WF1 initiated by phthalate. We specifically addressed whether all these adjuvants (1) induce phthalate-cross reactive anti-DNA response; (2) exacerbate these adverse effects following booster immunizations; and (3) affect host immune microenvironment in terms of systemic chemokines and cytokines.

## Methods

### Animals and Antigen

Female NZB/WF1 mice 8 weeks of age were purchased from Jackson laboratory and were housed in the animal facility of Indiana State University under a protocol approved by the Institutional Animal Care and Use Committee (IACUC) of Indiana State University. Ortho-phthalate-protein conjugates were prepared by azo-coupling of diazotized 4-aminophthalic acid (disodium) to KLH as described by Ghosh et al [22].

### Immunization Regimen

The phytol derivatives, PHIS-01 and PHIS-03 (US patent pending), were obtained by chemical modification of phytol according to the literature [23-26]. Suspensions of porcine small intestinal submucosa (SIS hydrate, SIS-H), a commercial biomaterial for surgical use were provided by Cook Biotech, Lafayette, IN. Alum was purchased from Sigma Chemical. The inocula consisted of 200 μL of phthalate-KLH (100 μg/mice) and equal volumes of either PHIS-01 (43 mg), PHIS-03 (5 mg), SIS-H (5 mg in 15% arlacel A, an emulsifier), or Alum. Doses were chosen based on our previous studies in BALB/c and C57BL/6 [8]. These ingredients were vigorously mixed a few times in a syringe and by vortexing. Inocula thus prepared were administered intra-peritoneally (i.p.) to six to eight-week old NZB/WF1 mice in a volume of 400 μL. Mice were given two booster injections at 10 day-intervals and bled 5 days after each immunization through retro-orbital veins. The parallel control groups of mice were immunized with only ortho-phthalate-KLH but no adjuvant. To determine how long the effects of immunizations would persist, the adjuvanted and control groups were administered with just phthalate-KLH, 5 months after the last immunization. Five days after this immunization, mice were bled, sera collected and assayed for antibody response.

### Assessment of Serum Levels and Isotype of Anti-Phthalate and Anti-DNA Antibodies

Serum anti-phthalate and anti-DNA antibody responses were determined in triplicates using enzyme linked immunosorbant assays (ELISA), as described previously [19]. Isotyping was done in triplicates using mice sera at 1/1000 dilution using ELISA plates coated with either phthalate-BSA or calf thymus DNA. Commercial isotype-specific rabbit antisera at 1:500 dilutions were used, and the assay was carried according to the manufacturer's protocol (Southern Biotech, Birmingham, AL).

### Assessment of Cytokine and Chemokines

Cytokine and chemokine profiles of control and experimental mice were assessed using mouse RayBiotech inflammatory cytokine array kits. Sera collected were diluted 1:5 in the reagent provided with the kits. Detection of cytokine was done according to the manufacturer's protocol. Membranes were exposed to X-ray films (Kodak X-OMAT AR film), and signal intensities were quantified and analyzed using Image J software from NCBI [27]. Biotin- positive and negative controls at six spots were used to normalize the results from different membranes. For each spot, the net optical density level was determined by subtraction of background density from the sample density and divided by the density of positive controls. The results were expressed as a percentage of relative intensity (RI) of experimental to positive control.

### Renal Pathologic Evaluation

At 8 months of age, mice were sacrificed, and urine and blood samples were collected. Blood urea nitrogen (BUN) and proteinuria were tested using Azostix and Multistix [19]. Proteinuria and BUN were estimated following the manufacturers' protocols. Kidneys tissues isolated were fixed in 4% paraformaldehyde. Slides were stained using hematoxylin and eosin (H&E). Histology was performed double-blindly at the laboratory of Dr. Roland M. Kohr M.D., Chief of Pathology and Certified Pathologist at the Terre Haute Regional Hospital.

### Statistical evaluation

One-way ANOVA was used to determine statistical significance compared to no-adjuvant treated group. Levels of p ≤ 0.05 were considered statistically significant. Data were expressed as mean ± SD.

## Results

### Effects of adjuvants on antibody response to phthalate in NZB/WF1 mice

**Figure 1:**
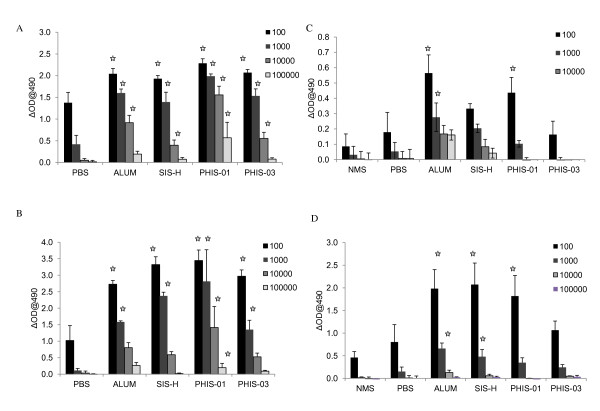
**Antibody response in autoimmune-prone NZB/WF1**. Mice immunized with phthalate-KLH Conjugate induce both anti-phthalate Antibody and anti-DNA Responses. The results represent sera of at least 5 mice tested individually using ELISA. A) anti-phthalate antibody levels after 2^nd ^booster immunization. B) Anti-DNA antibody levels after 2^nd ^booster immunization. C) anti-phthalate antibody levels after 3^rd ^booster immunization with antigen alone. D) Anti-DNA antibody levels after 3^rd ^booster immunization with antigen alone. The results represent mean from individual sera ± SD (n = 5 mice per group). The significance in experimental groups was determined relative to the group given antigen only (no adjuvant group) at the level of p ≤ 0.05.

Anti-phthalate antibody responses were induced in NZB/W F1 mice (8 weeks old) by repeated vaccination and sera assayed for evaluation of adjuvants. Groups of mice (n = 5) received two booster immunizations at 10- day intervals and then again after 5 months (by then the mice were 8 months old) of resting, and were injected with only phthalate-KLH but no adjuvant. Alongside, the control groups were exposed to phthalate-KLH without adjuvant. Results shown in Figure 1 (A, B) reveal that all mice immunized with adjuvanted phthalate-KLH developed significant levels of high-titer antibodies. However, the phytol derivative PHIS-01 enhanced antibody titer 10-fold over what was registered with SIS-H, PHIS-03, and alum. Moreover, only the adjuvanted groups, but not the control non-adjuvanted groups, responded with high-levels of serum anti-phthalate antibody following antigenic stimulation given after a period of five months. Among the adjuvanted groups, PHIS-01-treated group was the best responder in terms of specific antibody response, followed by alum. Responses in PHIS-03 and SIS-H groups were relatively less robust.

Immunization with phthalate-KLH has previously been shown to evoke cross-reactive antibody to self- ds-DNA [19]. We determined whether adjuvants could influence induction of this cross-reactivity that was previously reported to occur when mice were injected with phthalate-KLH and Diethyl hexyl phthalate (DEHP) [3]. The results in Figure 1 (C, D) show that mice immunized with alum-adsorbed phthalate-KLH developed significantly higher levels of anti-DNA response compared to phytol or SIS adjuvants after two booster immunizations (p ≤ 0.05). When all groups were rechallenged with phthalate-KLH after a 5-month period, they experienced an upswing in anti-DNA response that varied considerably among adjuvant-treated groups. The order of response in terms of titer was higher in alum and SIS-H groups, followed by PHIS-01 treated group. Mice immunized with phthalate-KLH emulsified in PHIS-03 developed less anti-DNA response.

### Effects of Adjuvants on antibody isotype profile

**Figure 2:**
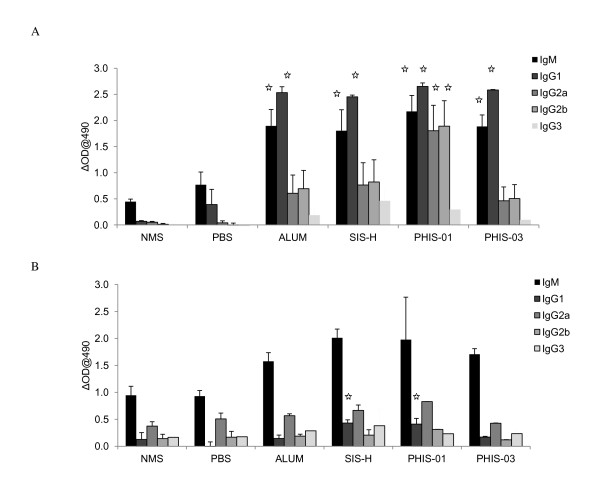
**Determination of IgG Sub-classes**. Determination of IgG sub-classes of (A) anti-phthalate antibodies and (B) anti-DNA induced with phthalate-KLH conjugates mixed in different adjuvants. The results represent sera of at least 5 mice tested individually using ELISA using commercial ELISA isotyping kits. The results represent mean from individual sera ± SD (n = 5 mice per group). The significance in experimental groups was determined relative to the group given antigen only (no adjuvant group) at the level of p ≤ 0.05

The quality of antibody response to both phthalate and ds-DNA in adjuvant-treated groups was further assessed in terms of isotype switching after repeated immunizations. The results in Figure 2 (A, B) compared the effectiveness of each adjuvant to modulate Ig isotypes induced following immunization with phthalate. However, in the adjuvanted groups, there was a clear indication of isotype switching, the most discernible one being IgG2a. In the absence of any adjuvant, phthalate-KLH conjugate could only induce a modest level of IgG sub-classes. All groups significantly induced major IgG sub-classes with the following trend in magnitude IgG1> IgG2b ≥ IgG2a ≥ IgG3. The relative ratios of (IgG2a+ IgG 2b+ IgG3)/IgG1 induced in response to alum and experimental adjuvants indicate that the specific antibodies elicited using alum, SIS-H and PHIS-03 are largely IgG1; this is suggestive of a Th2 bias (Figure 2A). Interestingly, PHIS-01was unlike the other adjuvant-treated groups and developed significantly high IgG2a and IgG2b levels directed to phthalate (Figure 2A). While the isotype profile of anti-phthalate response was marked by an increase in IgG subclass, the anti-DNA response induced consisted mostly of IgM class with only low levels of IgG subclasses (Figure 2B).

### Changes in chemokine microenvironment due to adjuvants

**Figure 3:**
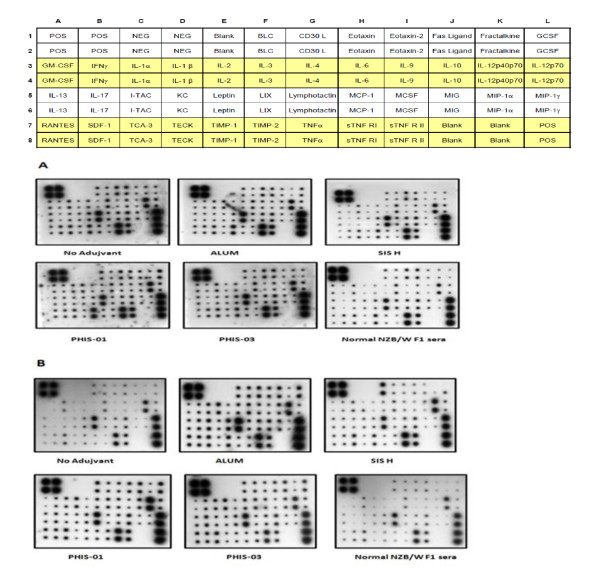
**Systemic cytokine and chemokine profiles in NZB/WF1 mice immunized with phthalate-KLH**. NZB/WF1 mice (N = 5) were immunized as described under Materials and Methods, and sera (collected after 2^nd ^booster, and then the 3^rd ^booster with the antigen phthalate-KLH alone) were diluted 1:5 and subjected to cytokine and chemokine antibody arrays. Each cytokine was measured in duplicate spots and displayed in the array template as shown. The image shown in (A) represents serum cytokine and chemokine profiles of mice treated with different adjuvants in combination with antigen after receiving two booster immunizations, and the image shown in (B) represents serum cytokine and chemokine profiles of mice treated with different adjuvants in combination with the antigen following the 3^rd ^booster immunization with antigen alone.

**Figure 4:**
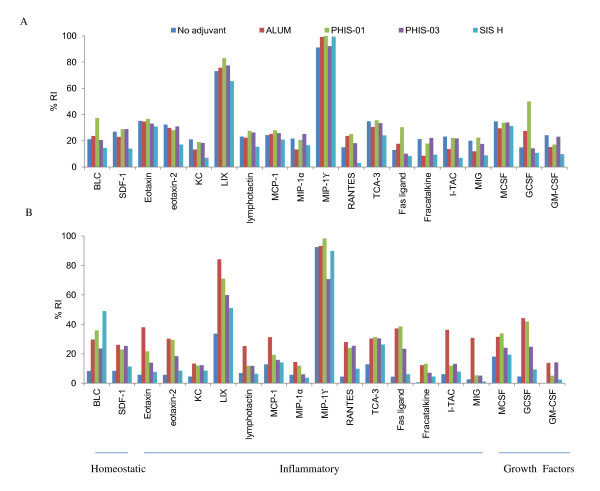
**Systemic chemokine profiles in NZB/W F1 mice immunized with phthalate-KLH**. NZB/W F1 mice (N = 5) were immunized as described under Materials and Methods, and sera collected (after 2^nd ^booster, and then the 3^rd ^booster with the antigen phthalate-KLH alone) were diluted 1:5 and subjected to cytokine and chemokine antibody arrays. Each cytokine was measured in duplicate spots and displayed in the array template as shown. Densities of each spot from images shown in Figure 3 were semi-quantified using image J software [27] and expressed as relative units to positive controls provided with the array. The image in (A) represents chemokine profiles in sera of mice immunized 3 times with the antigen and adjuvants, and the image shown in (B) represents chemokine profiles of mice treated receiving a 3^rd ^booster immunization with antigen alone.

Chemokines are considered important regulators of innate immunity. It is very likely that adjuvant efficacy lies in their ability to induce chemotactic factors and pro-inflammatory cytokines, which regulate the interplay and cross-talk between innate and acquired immune systems. As shown in Figure 3 and analyzed in Figure 4, treatments with phthalate KLH alone or in combination with different adjuvants resulted in induction of clusters of chemotactic factors, which are known to be responsible for recruitment of cells belonging to innate immunity, namely, neutrophils, monocytes, macrophages and B1 cells. Analyses of Figure 4A show that LIX and MIP-γ (high expression,) MCP-1, lymphotactin, SDF-1, MCSF, Eotaxin, Eotaxin 2, KC, I-TAC, and MIG (medium expression ) were induced by antigen alone and also in combination with either ALUM, or phytol derivatives PHIS-01 and PHIS-03. The SIS-treated group also expressed the same cytokines but less pronounced. Interestingly, PHIS-01 induced more BLC, G-CSF and FAS ligand. Later after 5 months, when all adjuvanted and control mice groups received phthalate KLH alone, the chemokine profiles (Figure 4B) differed among groups in magnitude. Mice receiving only phthalate-KLH regimen expressed only LIX and MIP-1γ, which seem to be constitutive in NZB/WF1 mice. Whereas PHIS-01, PHIS-03, and SIS-H down- regulated the expression of MIG and TIMP-1, alum augmented expressions of I-TAC, G-CSF, Eotaxin, MIG, lymphotactin and MCP-1 (Figure 4). Interestingly, mice receiving SIS-H upregulated the expression of BLC, suggesting an increase in involvement of B1 cells.

### Changes in cytokine microenvironment due to adjuvants

Immunogenic stimulation changes local cytokine milieu that in turn helps to recruit specific T helper and different effector cells. Cytokines IFN-γ, IL-2, IL-12, and G-CSF promotes T-helper-mediated reactions and stimulates strong cellular immune responses, while IL-3, -4, -5, -6, -10, and IL-13 brings out Th2 response favoring specific humoral response. The hallmark of lupus could be due to an imbalance between Th1 and Th2-related cytokines [28]. In order to assess the cytokine profile induced in immunized mice, we collected sera after 2nd booster immunizations from all experimental and non-adjuvanted control groups. We also assessed sera from mice given a 3^rd ^booster immunization with phthalate-KLH alone.

**Figure 5:**
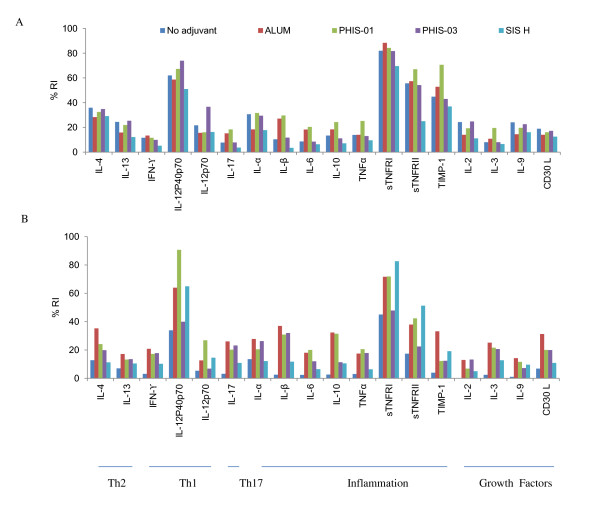
**Systemic cytokines profiles of NZB/W F1 mice immunized with phthalate-KLH**. NZB/W F1 mice (N = 5) were immunized as described in materials and methods, and sera collected after 2^nd^, and 3^rd ^booster immunizations were diluted 1:5 and subjected to cytokine and chemokine antibody arrays. Each cytokine is represented by duplicate spots in the array as shown in the array template. Densities of each spot from images shown in figure 3 were semi-quantified using image J software and expressed as relative units to positive controls provided in the array. The image in (A) represents cytokine profiles in sera of mice immunized 3 times with the antigen and adjuvants, and the image shown in (B) represents cytokine profiles of mice treated receiving a 3^rd ^booster immunization with antigen alone.

Results of this study are given in Figure 3 and analyzed in Figure 5. As shown in Figure 5A, NZB/W F1 mice immunized with phthalate KLH alone or with adjuvants as well registered moderate expressions of cytokines that modulate both Th1 (due to IFN-γ, IL-12) and Th2 (due to IL-3, -4, -10, and -13) populations, and a low but detectable levels of pro-inflammatory cytokines associated with inflammatory response (IL-1, IL-6, IL-17, TNF-α). Alum and PHIS-01 produced an upward trend in IL-1β, IL-17, IL-6, and IL-10, while PHIS-03 induced more of IL-12 P70. All adjuvant-treated groups induced high levels of anti-inflammatory cytokines TNFR II, TNFR I, and TIMP1; also induced a high level of IL-4 compared to INF-γ which may suggest Th2 dominance. Expression of cytokines described above was less pronounced in groups treated with SIS-H.

Interestingly, five months after the 2^nd ^booster immunization, NZB/WF1 mice that received only phthalate KLH had significantly lower expressions of cytokines tested than those in adjuvanted groups. Clearly, adjuvants in vaccine formulations made a difference. Adjuvants magnified antibody response and conferred longer memory of this response brought about with changes in chemokine/cytokine microenvironment. During the 32-week post-immunization period, PHIS-03 induced increased expressions of IL-1β, INF-γ, IL-3, IL-17 and TNFα to levels similar to those induced by PHIS-01 or alum. However, there was no significant change noticed on the level of these cytokine in mice treated with SIS-H. It is noteworthy that PHIS-03 caused down-regulation of TNFR I, TNFR II, and IL-12 P40P70, whereas SIS-H, alum and PHIS-01 did not. All adjuvant-treated groups, however, down regulated the expression of TIMP-1.

### Signs of Nephritis

**Figure 6:**
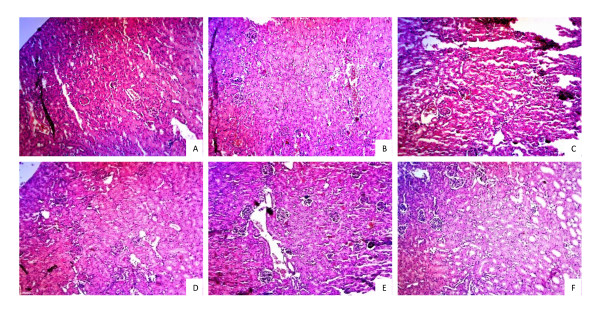
**Renal histopathology**. Kidney tissues from following six groups of NZB/W F1 mice previously described were harvested at 8 months of age and examined as described in materials and methods. Representative H&E stained kidneys (magnification ×100 ) are shown as: Group A: age matched mice; Group B: Mice immunized with phthalate-KLH alone; Group C: Mice immunized with phthalate-KLH adsorbed to alum; Group D: Mice immunized with phthalate-KLH emulsified with PHIS-01; Group E: Mice immunized with phthalate-KLH emulsified with PHIS-03; Group F: Mice immunized with phthalate-KLH emulsified with SIS-H.

**Figure 7:**
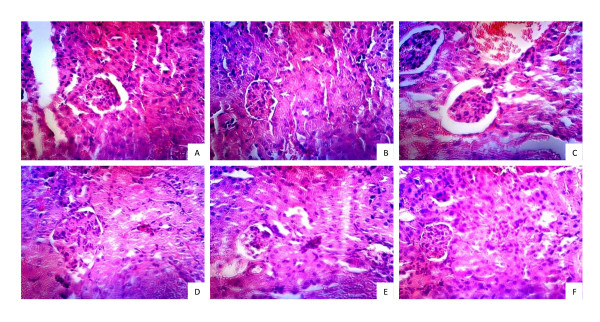
**Renal histopathology**. Kidney tissues from following six groups of NZB/W F1 mice previously described were harvested at 8 months of age and examined as described in materials and methods. Representative H&E stained kidneys (magnificationx400) are shown as: Group A: age matched mice; Group B: Mice immunized with phthalate-KLH alone; Group C: Mice immunized with phthalate-KLH adsorbed to alum; Group D: Mice immunized with phthalate-KLH emulsified with PHIS-01; Group E: Mice immunized with phthalate-KLH emulsified with PHIS-03; Group F: Mice immunized with phthalate-KLH emulsified with SIS-H.

Repeated immunizations of NZB/WF1 mice with phthalate-KLH plus different adjuvants resulted in significantly higher anti-phthalate response but the anti-DNA response varied among adjuvant-treated groups. In order to determine whether any clinical signs of nephritis were evident following development of phthalate-induced anti-DNA antibody, we determined urinary protein and blood urea nitrogen (BUN) levels in all NZB/WF1 groups at 8 months of age. Results in Table 1 revealed that mice treated with phthalate-KLH plus alum or SIS-H had higher levels of anti-DNA antibodies than in PHIS-01, or PHIS-03. On examination of kidney tissues for histopathological changes in a double blind fashion, no major changes were noted in connective or glomerular tissues between untreated or adjuvant-treated groups (Figures 6 and 7). We primarily detected different levels of lymphoid infiltration. Groups treated with alum, PHIS-01, PHIS-03, and SIS-H had medium lymphoid aggregates.

**Table 1:** Assessment of clinical parameters of kidney pathology

	NZB/W F1 immunized with Phthalate- KLH in combination with different adjuvants					
	
	Untreated mice	No adjuvant	ALUM	PHIS-01	PHIS-03	SIS-H
Proteinura (mg/dL)	0.3	126	166.7	132	47.5	232

BUN (mg/dL)	15	39.6	53.33	46.5	23.25	56.5

These results suggest that while treatments with phthalate-KLH in combination with adjuvants increased anti-DNA levels, no severe signs of nephritis or abnormality in kidney tissue were observed. Furthermore, mice did not die prematurely in all adjuvant-treated groups except in alum-treated group, where 2 out 5 mice treated died at 8 months of age, and only 1 mouse out of 5 died in untreated mice as well as in those treated with SIS-H or PHIS-01. However, there was no mortality in mice treated only with phthalate-KLH alone or in combination with PHIS-03. The average life span of female NZB/WF1 mice is 245 days.

## Discussion

During this study, we hypothesized that appropriate adjuvants can alter host microenvironment, specifically the cytokine milieu; this may bestow ameliorating effects by changing the course of immune response. Previously, we showed that phthalate as a conjugate or as DEHP (diethyl hexyl phthalate, a plasticizer) can induce cross reactive anti-DNA antibody response, and promote lupus-like syndromes in NZB/WF1 mice [19-21]. The ability of phthalate to induce both anti-phthalate and cross reactive anti-DNA antibodies was, therefore, utilized to assess the efficacy and safety of novel adjuvants, PHIS-01, PHIS-03, and SIS-H relative to alum. We used alum as a reference adjuvant since it is the only human-licensed adjuvant and in many respects similar to experimental adjuvants tested in this study. Anti-phthalate IgG subclasses induced as a result of these adjuvants suggests a bias towards the Th2 response. Such a response typically is helpful primarily to combat extracellular bacteria, but it may be beneficial against intracellular bacteria like Francisella tularensis (33). However, the role of antibodies in protection is better documented against extracellular than intracellular pathogens.

Phytol-based adjuvants and SIS biomaterials seem to be as effective as alum in enhancing anti-phthalate antibody response. However, their impacts differed with respect to inducement of cross reactive anti-ds-DNA response with the ageing of the mice. In 3 months old NZB/W F1 mice, the response to phthalate culminates in high-titer anti-phthalate antibodies in all adjuvanted groups. Relatively, PHIS-03 treated group registered the lowest anti-ds-DNA response. Overall, anti-DNA response in all cases is of low titer, low-affinity IgM type antibody which is not linked to pathogenicity or aggravation of lupus-like diseases [29-31]. In contrast, high affinity IgG2a and IgG3 anti-DNA antibody classes are considered pathogenic, because they aggravate lupus-like diseases [31,32]. Furthermore, there are reports demonstrating that when IgM autoantibodies are induced, they reduce the severity of autoimmune pathology due to IgG autoantibodies [31]. We have observed that as the NZB/WF1 mice grow older, by the eighth month their serum levels of Ig2a and other IgG isotypes are higher in antigen and alum-treated groups compared to those treated with only the experimental phytol-based adjuvants. Previous reports by Lim et al. also showed that inclusion of CFA/IFA in vaccine formulations containing phthalate KLH increases IgG2a anti-DNA antibody, which correlates with lupus-like symptoms in treated NZB/WF1mice [21]. The results suggest that, our experimental adjuvants are superior to the alum group.

The above assessment is also supported by cytokine/chemokine. Cytokines and chemokines play an essential role in the outcome of immune response [33]. The profiles of chemokines, proinflammatory cytokines, and Th1/Th2 ratios assessed in this study clearly establish that PHIS and SIS compounds have ameliorating effects as adjuvants. Both alum and new adjuvants are capable of inducing chemokines such as LIX, BLC, MCP-1, RANTES, and Eotaxin. These chemokines are necessary for the recruitment of neutrophils, monocytes, macrophages and immature dendritic cells (iDC), as well as B cells; all these cells play important roles in uptake of antigen and subsequent development of adaptive response [34]. However, over-expression of chemokines such as MCP-1, RANTES, or BLC has been linked to lupus nephritis in patients and in animal models of the disease [35-39]. But at 8 months of age and despite repeated exposure to phthalate, all adjuvanted groups promote only moderate levels of these chemokines.

Overall, only limited pro-inflammatory response has been observed in all groups. The pro-inflammatory response in terms of IL-1α, IL-1β, IL-6 and TNF-α due to PHIS-01 and PHIS-03 is similar to that of alum. The SIS-H biomaterial is even better; they provoke no measurable pro-inflammatory cytokines. Interestingly, all adjuvants can cause moderate lymphocyte infiltration as shown in kidney histology.

Infiltration of neutrophils and monocytes to kidney is known to correlate with proteinuria and onset of kidney damage [40,41]. There is virtually no onset of renal pathology in mice that are 8-month old. This is unlike what has been reported about Freunds' adjuvants and squalene in MF59 [12,19,42,43]. Possibly the explanation lies in IL-10, which actually goes up and hinders pro-inflammatory forces. Alum and PHIS-01 cause marked increase in IL-10 expression balancing the effect caused by pro-inflammatory cytokines. However, this is not the only reason for the outcome of immune response to phthalate triggered by each adjuvant.

Another hallmark of lupus is the imbalance of Th1/Th2 cytokines [28]. In a previous study, it has been shown that hydrocarbon oil adjuvant like pristane can induce an overproduction of Th1 polarizing cytokines like INF-γ [28]. This, together with high levels of IL-6 and TNF-α, can aggravate lupus-like diseases in rodents [42]. In our study, alum, PHIS-01 and PHIS-03 can induce more Th1 and Th2 cytokines than SIS-H. In addition, PHIS-01 is very effective in inducing IL-12, and this is the only cytokine that can facilitate production of significant amount of IgG2a subclass signifying a shift towards Th1. Interestingly, the Th1 and Th2 responses generated by different adjuvants is directed more toward phthalate as is evident by induction of IgG subclass, whereas the cross reactive anti-ds DNA response is mostly IgM with little IgG subclass switching. This ability of adjuvants to selectively activate antigen-specific T cells without provoking auto-reactive class-switching T cells is of great interest in adjuvant design. It is worthwhile to assess also the efficacy of adjuvants at the level of antibody gene repertoire, especially at the level of the antibody light chain repertoire. As previously documented in several reports, induction of specific light chain-like V kappa1 genes greatly increases the pathogenic properties of autoantibodies produced during autoimmune response [44,45]. Our ongoing study would focus on characterization of antibody light chain repertoire induced by different adjuvants and its significance on suppression or aggravation of phthalate induced lupus like autoimmune response.

## Conclusion

In conclusion, alum, SIS-H, or phytol compounds do not engender lupus-like syndromes in NZB/WF1 mice. They appear to be safe and highly effective. No deleterious effects of physiological significance result from the use of experimental adjuvants described here. In this regard, these adjuvants are superior to other oil-in-water adjuvants like CFA/IFA or isoprenoids like pristane.

## Abbreviations

ECM: Extracellular matrix; SIS: Porcine small intestinal submucosa; PHIS: Phytol-based immune-stimulant; KLH: Keyhole limpet hemocyanin; ELISA: Enzyme linked immunosorbant assay; CFA: Complete Freund's adjuvant; IFA: Incomplete Freund's adjuvant; PBS: Phosphate buffer saline; i.p: Intra-peritoneal; BUN: Blood urea nitrogen; DEHP: Diethyl hexyl phthalate; iDC: Immature dendritic cells.

## Competing interests

The authors declare that they have no competing interests.

## Authors' contributions

YA and SKG both conceived and designed the experimental approach and method of study. YA and SKG executed the experiments and drafted the manuscript. YA and SKG analyzed the data and critically revised the manuscript.
